# The Prognostic Relevance of PMCA4 Expression in Melanoma: Gender Specificity and Implications for Immune Checkpoint Inhibition

**DOI:** 10.3390/ijms23063324

**Published:** 2022-03-19

**Authors:** Luca Hegedüs, Elisabeth Livingstone, Ágnes Bánkfalvi, Jan Viehof, Ágnes Enyedi, Ágnes Bilecz, Balázs Győrffy, Marcell Baranyi, Anna-Mária Tőkés, Jeovanis Gil, György Marko-Varga, Klaus G. Griewank, Lisa Zimmer, Renáta Váraljai, Antje Sucker, Anne Zaremba, Dirk Schadendorf, Clemens Aigner, Balázs Hegedüs

**Affiliations:** 1 Department of Thoracic Surgery, University Medicine Essen–Ruhrlandklinik, 45239 Essen, Germany; luca.hegedues@rlk.uk-essen.de (L.H.); jan.viehof@rlk.uk-essen.de (J.V.); clemens.aigner@rlk.uk-essen.de (C.A.); 2 Department of Dermatology, University Medicine Essen, 45147 Essen, Germany; elisabeth.livingstone@uk-essen.de (E.L.); klaus.griewank@uk-essen.de (K.G.G.); lisa.zimmer@uk-essen.de (L.Z.); renata.varaljai@uk-essen.de (R.V.); antje.sucker@uk-essen.de (A.S.); anne.zaremba@uk-essen.de (A.Z.); dirk.schadendorf@uk-essen.de (D.S.); 3 Department of Pathology, University Medicine Essen, 45147 Essen, Germany; agnes.bankfalvi@uk-essen.de; 4 Department of Transfusiology, Semmelweis University, 1085 Budapest, Hungary; enyedi.agnes@med.semmelweis-univ.hu; 5 2nd Department of Pathology, Semmelweis University, 1085 Budapest, Hungary; bilecz.agnes@med.semmelweis-univ.hu (Á.B.); baranyi.marcell@med.semmelweis-univ.hu (M.B.); tokes.anna_maria@med.semmelweis.univ.hu (A.-M.T.); 6 Department of Bioinformatics, Semmelweis University, 1085 Budapest, Hungary; gyorffy.balazs@med.semmelweis-univ.hu; 7 Division of Oncology, Department of Clinical Sciences Lund, Lund University, 221 00 Lund, Sweden; jeovanis.gil_valdes@med.lu.se; 8 Clinical Protein Science & Imaging, Biomedical Centre, Department of Biomedical Engineering, Lund University, 221 00 Lund, Sweden; gyorgy.marko-varga@bme.lth.se

**Keywords:** melanoma, plasma membrane calcium ATPase 4, lung metastasis, immune checkpoint inhibition

## Abstract

PMCA4 is a critical regulator of Ca^2+^ homeostasis in mammalian cells. While its biological and prognostic relevance in several cancer types has already been demonstrated, only preclinical investigations suggested a metastasis suppressor function in melanoma. Therefore, we studied the expression pattern of PMCA4 in human skin, nevus, as well as in primary and metastatic melanoma using immunohistochemistry. Furthermore, we analyzed the prognostic power of PMCA4 mRNA levels in cutaneous melanoma both at the non-metastatic stage as well as after PD-1 blockade in advanced disease. PMCA4 localizes to the plasma membrane in a differentiation dependent manner in human skin and mucosa, while nevus cells showed no plasma membrane staining. In contrast, primary cutaneous, choroidal and conjunctival melanoma cells showed specific plasma membrane localization of PMCA4 with a wide range of intensities. Analyzing the TCGA cohort, PMCA4 mRNA levels showed a gender specific prognostic impact in stage I–III melanoma. Female patients with high transcript levels had a significantly longer progression-free survival. Melanoma cell specific PMCA4 protein expression is associated with anaplasticity in melanoma lung metastasis but had no impact on survival after lung metastasectomy. Importantly, high PMCA4 transcript levels derived from RNA-seq of cutaneous melanoma are associated with significantly longer overall survival after PD-1 blockade. In summary, we demonstrated that human melanoma cells express PMCA4 and PMCA4 transcript levels carry prognostic information in a gender specific manner.

## 1. Introduction

Melanoma is a rare form of skin cancer; however, its incidence continuously increases in the Western World [1]. While patients with a thin primary melanoma have a favorable prognosis, the 5-year survival rate of patients with metastatic disease is below 30% [2]. Cutaneous melanoma develops from melanocytes that reside in the basal layer of the skin where they produce and transfer melanin to the surrounding keratinocytes to provide protection from UV radiation. Benign melanocytic proliferations can also appear within the layers of the skin. Interestingly, BRAF V600E mutations are present at high frequency in nevi and probably serve as an initiating event for nevi formation [3]. Dysplastic nevi are irregular in their pigmentation, shape and borders. They can evolve from common nevi or de novo and have a higher mutational burden [4]. Malignant cutaneous melanoma can originate either from a nevus or de novo in chronically sun-damaged areas (CSD) or in regions with less sun exposure. Non-CSD melanomas develop typically in younger patients and predominantly carry *BRAF* mutations, while CSD melanomas have a higher mutation burden, more often contain *NF1*, *NRAS* or *KIT* mutations and affect older people at the distal extremities and head and neck region [5,6]. Distant metastases of cutaneous melanoma develop primarily in the lung, brain, liver and bone. Furthermore, melanocytes are also present in the uveal tract including the iris, ciliary body and choroid and are the cells of origin for uveal melanoma. In uveal melanoma the mutational burden is low and *GNA11*, *BAP1* and *SF3B1* genes are frequently mutated [7].

Intracellular calcium, as a second messenger, plays an important role in the regulation of essential cellular processes such as proliferation, cell migration or cell death. In cancer cells, Ca^2+^ homeostasis is altered due to the remodeling of the expression and activity of calcium regulatory proteins such as Ca^2+^ channels and transporters [8]. Plasma membrane Ca^2+^ ATPases (PMCA) maintain a low intracellular calcium concentration by removing excess calcium from the cytosol. There are four PMCA genes (called *ATP2B1-4*) coding for the four proteins PMCA1, PMCA2, PMCA3 and PMCA4, from which more than 20 isoforms are generated through alternative splicing [9]. Apart from PMCA1b, which is the housekeeping isoform, PMCA4b is also ubiquitously expressed. Alterations in its expression and activity were described in several cancer types including breast, pancreatic and colon cancer. Analysis of breast cancer cell lines showed a subtype specific expression of PMCA4b with decreased expression in estrogen receptor (ER) positive cells but not in triple negative cell lines. In ER positive cells, PMCA4b expression increased after histone deacetylase inhibitor treatment and in MCF-7 cells also by treatment with 17β-estradiol, probably by increasing transcription through a putative ER-α binding site in the *ATP2B4* gene [10,11]. Analysis of colon cancer tissues showed that PMCA4 protein expression gradually decreases from high-grade adenoma to colon carcinoma to lymph node metastasis samples compared to normal mucosa where PMCA4 is abundantly present. Interestingly, the mRNA levels of PMCA4 were not altered in the same samples [12]. Recently, gene expression data analysis revealed that in pancreatic ductal adenocarcinoma, PMCA4 expression increased compared to healthy tissue and this correlates with worse patient survival. Downregulation of PMCA4 expression in pancreatic ductal adenocarcinoma cell lines reduced the wound healing capacity of the cells and sensitized them to apoptotic stimuli [13].

Previously, we investigated the role of PMCA proteins in the regulation of intracellular calcium signaling in melanoma cell lines. We found that two PMCA proteins, PMCA1 and PMCA4b, were present in melanoma cells, however, the expression of PMCA4b was downregulated in BRAF mutated cell lines. Inhibition of the BRAF/MEK/ERK pathway increased the abundance of PMCA4b, but not of PMCA, in BRAF mutated cells and this was coupled with enhanced calcium clearance, decreased cell motility in vitro and reduced metastatic capacity in vivo [14]. We also demonstrated that the expression of PMCA4b and PMCA1 is under epigenetic control in melanoma cell lines while histone deacetylase (HDAC) inhibitor treatment increased the abundance of both proteins. The increased PMCA4b level after HDAC inhibition strongly reduced cell migration of A375 BRAF mutated melanoma cells [15]. Our data revealed that PMCA4b is a metastasis suppressor as it strongly inhibited the migration and metastatic capacity of BRAF mutated cells, but it did not affect cell growth. Further analysis showed that reduced expression of PMCA4b in BRAF mutated melanoma cells is partially due to the increased degradation of PMCA4b induced by elevated activity of the p38 MAPK pathway. Inhibition of this pathway increased the abundance of PMCA4b by increasing its stability [16]. Finally, PMCA4b was able to inhibit cell migration through the remodeling of the actin cytoskeleton by increasing cell–cell connections as well as lamellipodia and stress fiber formation [17].

In the last decade, the treatment of metastatic melanoma has undergone a revolutionary transformation as a result of the emergence of molecularly targeted inhibitors and the introduction of immune checkpoint inhibition [18,19,20,21]. Due to the high mutational burden and immunogenicity in melanoma, these tumors are excellent targets for the manipulation of tumor infiltrating lymphocytes [22]. For this very reason, immune checkpoint inhibitor (ICI) monoclonal antibodies against cytotoxic T-lymphocyte antigen-4 (CTLA-4) and programmed death-1 (PD-1) are now the first line treatment for metastatic melanoma [23]. While predictive molecular biomarkers are available for the targeted therapy of specific driver mutations, there is still an urgent need for predictive biomarkers that can identify the patients who can benefit the most from immune checkpoint inhibition [24].

Based on earlier preclinical findings suggesting a role for PMCA4 in melanoma progression, in the present study, we explored the expression of PMCA4 in human skin, nevi, primary melanomas and lung metastasis on the protein level. After demonstrating the expression of PMCA4 in melanoma cells, we also studied the association of PMCA4 mRNA and protein levels with disease outcome and PD-1 blockade therapy response.

## 2. Results

### 2.1. Differentiation Specific Expression of PMCA4 in Human Epithelia

**Figure 1:**
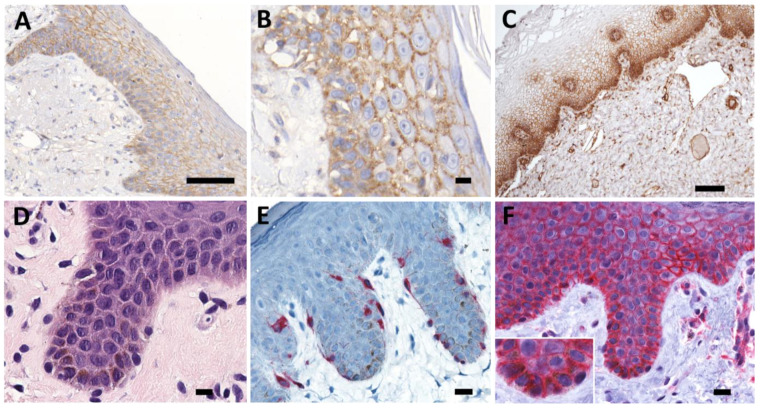
PMCA4b expression in human skin and mucosa. (**A**,**B**) In the skin, PMCA4b expression is increasing from the basal cells to the stratum spinosum and granulosum. The PMCA4b staining pattern resembles desmosomal localization. (**C**) In the vaginal mucosa, basal cells have the highest PMCA4b expression. (**D**–**F**) The hematoxylin-eosin staining of the vulval epidermis shows a high number of pigment containing cells. (**E**) Melan-A staining (red) identifies the melanocytes in the basal cell layer that are characterized by a distinct nuclear morphology. (**F**) The plasma membrane between melanocytes and basal cells stained for PMCA4, whether the proteins are localized on the melanocytic plasma membrane as well cannot be unequivocally determined. Scale bars: (**A**,**C**): 100 µm; (**B**,**D**–**F**): 10 µm.

As there are no reports on the abundance and localization of PMCA4b protein in the human skin, we performed immunohistochemical analysis of skin and vaginal mucosa specimens and showed that skin keratinocytes express PMCA4 in a differentiation dependent manner (Figure 1A–C). Basal cells demonstrated a relatively lower expression, while cells in the stratum spinosum and granulosum had a higher and stronger membrane specific expression. Especially in the stratum spinosum, the staining pattern suggests co-localization with desmosomes (Figure 1B). Interestingly, in vaginal mucosa, the basal cell layer has the strongest PMCA4b expression and it is gradually decreasing towards the mucosal surface (Figure 1C).

### 2.2. PMCA4b Expression in Nevus

**Figure 2:**
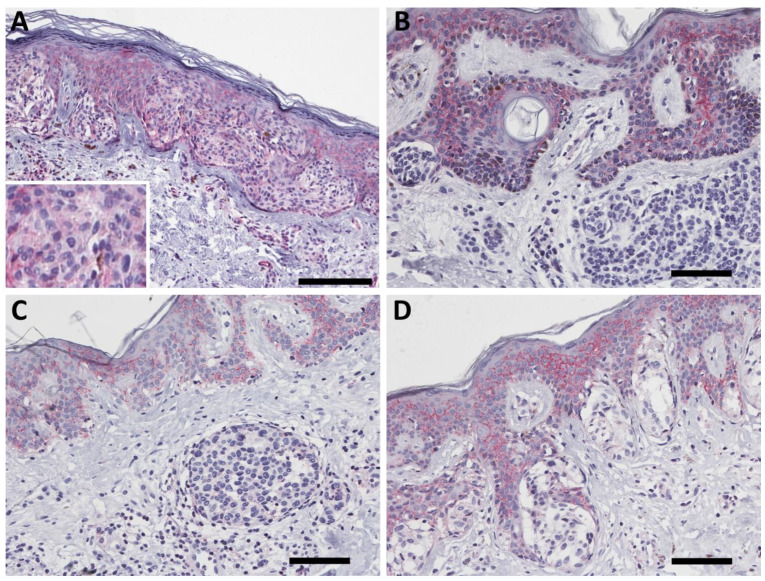
PMCA4b expression in human nevus. No plasma membrane specific staining was observed in any of the nevi (**A**—junctional, **B**—compound, **C**—intradermal and **D**—dysplastic). Scale bars: (**A**) 100; µm; (**B**–**D**): 50 µm.

To study whether the PMCA4b expression is altered during nevus development, we stained a series of 26 nevi including junctional, compound and dermal nevi as well as a panel of dysplastic nevus (Supplementary Materials Table S1 and Figure 2). Interestingly, we found no plasma membrane specific staining of PMCA4b in human nevus specimens.

### 2.3. PMCA4b Expression in Primary Melanoma

**Figure 3:**
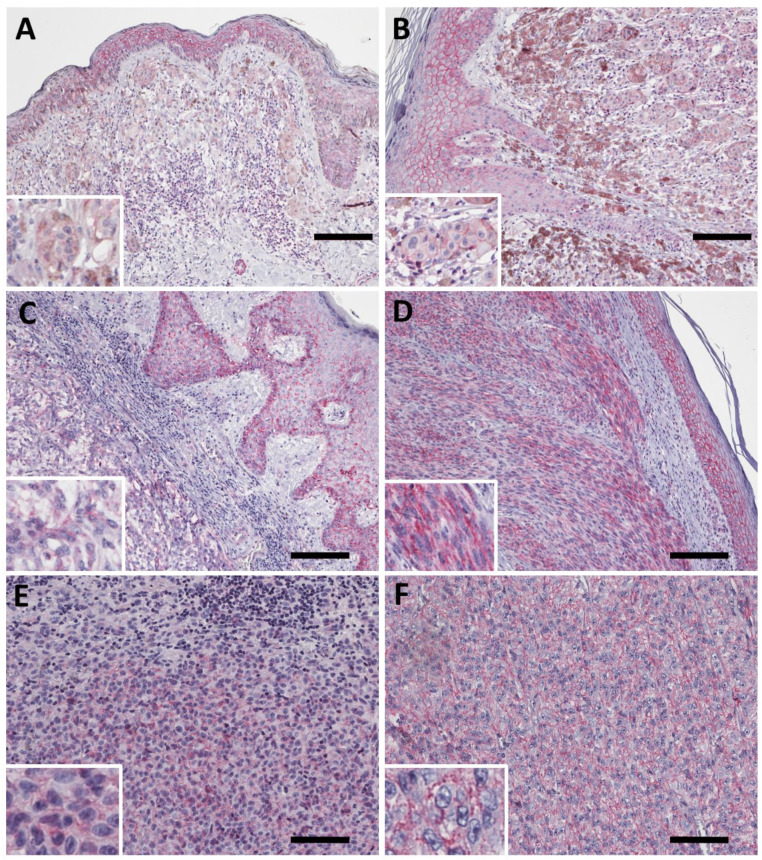
Representative images of PMCA4b expression in human primary melanoma. (**A**) Weak but clear plasma membrane-specific PMCA4b expression in cutaneous melanoma. (**B**,**C**) Intermediate staining intensity in epitheloid (**B**) and mixed morphology (**C**). (**D**) High PMCA4b expression in a cutaneous melanoma with spindle cell morphology. (**E**,**F**) Plasma membrane specific PMCA4b expression in conjunctival (**E**) and choroidal (**F**) melanoma. Scale bars: (**A**–**D**): 100 µm; (**E**,**F**): 50 µm.

To explore the expression of PMCA4b in human melanoma we stained primary cutaneous, choroidal and conjunctival melanoma specimens (Figure 3 and Table 1). In primary melanoma cases, there was a wide range of expression up to the high levels of PMCA4b expression that we observed in the epidermis (Figure 3).

The characteristic plasma membrane specific staining is present both in epitheloid and spindle cell morphologies among cutaneous melanoma samples. Furthermore, conjunctival and choroidal melanomas also expressed PMCA4 (Figure 3E,F).

### 2.4. PMCA4 mRNA Level Is a Gender-Specific Prognosticator in Primary Cutaneous Melanoma

**Figure 4:**
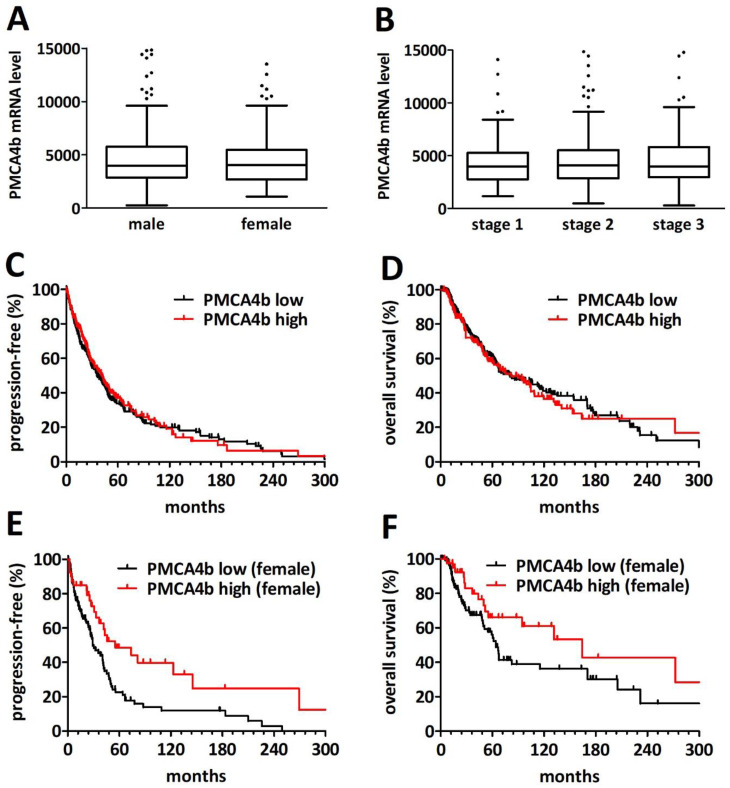
PMCA4 is a gender specific prognostic factor in the TCGA cohort of non-metastatic cutaneous melanoma patients. (**A**) There is no difference in the distribution of PMCA4 mRNA levels between male and female patients. (**B**) PMCA4 mRNA levels are independent of disease stage. (**C**,**D**) PMCA4 levels show no association with progression-free (**C**) or overall (**D**) survival in the overall cohort. (**E**) In female patients, high PMCA4 levels confer a significantly longer progression-free survival (*p* = 0.0037). (**F**) The difference in overall survival in female patients between high and low PMCA4 mRNA levels did not reach statistical significance (*p* = 0.0717).

To investigate the potential prognostic value of PMCA4b expression in cutaneous melanoma we analyzed the TCGA cutaneous melanoma database. We excluded the small number of stage IV/metastatic patients. The basic clinicopathological characteristics of the analyzed cohort are provided in Table 2. None of the investigated parameters—age, gender, site, disease stage or BRAF/NRAS mutation status—showed an association with PMCA4 mRNA levels (Figure 4A-B). Age, site and stage were significant prognosticators for both progression-free and overall survival (Supplementary Materials Figure S1).

PMCA4 mRNA levels showed no prognostic impact on the overall cohort (Figure 4C,D, for progression-free survival *p* = 0.657 and for overall survival *p* = 0.623). However, we found a significantly increased progression-free survival of the female patients with primary cutaneous melanoma with high (n = 41) PMCA4 mRNA levels when compared to low (n = 116) transcript levels (55 versus 29 months, *p* = 0.0037). A similar—albeit not significant—tendency was observed for overall survival (164 vs. 64 months, *p* = 0.072). Of note, using the Gehan–Breslow–Wilcoxon test which puts more weight on the early events, the difference reached significance (*p* = 0.047). In contrast, there was no significant difference in overall or progression-free survival in male patients when dichotomized by PMCA4 transcript levels (*p* = 0.218 and 0.176, Supplementary Materials Figure S2). Of note, PMCA1 mRNA levels showed a weak albeit significant positive correlation with PMCA4 transcript levels (Spearman r = 0.149, *p* = 0.0021; Supplementary Materials Figure S3).

### 2.5. PMCA4b Expression Is Decreased in Pulmonary Melanoma Metastasis with Anaplasticity but Has No Prognostic Impact after Metastasectomy

Next, we analyzed the expression of PMCA4 in melanoma lung metastasis using immunohistochemistry on FFPE specimens from our previously published lung metastasectomy cohort [25]. The melanoma cell specific scores were determined as: 0—no expression, 1, 2 and 3 for low, intermediate and high staining intensity. The epitheloid or spindle cell morphology as well as the presence of anaplastic components was also determined for each specimen.

**Figure 5:**
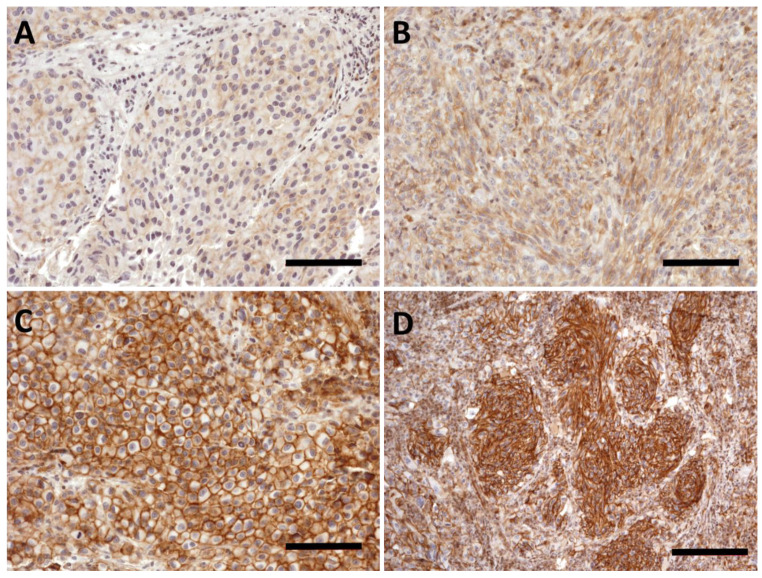
PMCA4b expression in melanoma lung metastasis. (**A**) Weak but clear plasma membrane-specific PMCA4b staining in a lung metastasis. (**B**) Intermediate intensity staining. (**C**,**D**) High PMCA4b expression in epitheloid (**C**) and spindle cell (**D**) melanoma. Scale bar: (**A**–**C**): 100 µm; (**D**): 200 µm.

We found a wide range of expression intensity in the lung metastasis specimens (Figure 5). In some cases, we found weak plasma membrane specific PMCA4b staining in melanoma cells (Figure 5A). When dichotomized for high (IHC score 2 to 3) and low expression (IHC score 0.5 to 1.5) we found no difference based on gender, age, maximum nodule size or BRAF/NRAS mutation status (Table 3).

**Figure 6:**
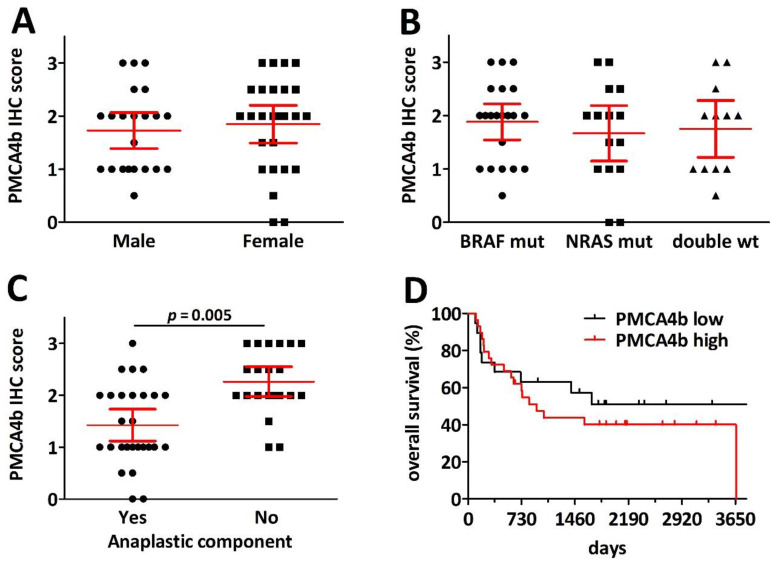
Clinicopathological analysis of PMCA4b protein levels in melanoma lung metastasis. (**A**) There is no difference in the distribution of PMCA4b expression between male and female patients. (**B**) BRAF and NRAS mutation has no impact on PMCA4b staining intensity. (**C**,**D**) Melanoma specimens with anaplastic components showed decreased PMCA4b expression. (**D**) There is no prognostic impact of melanoma cell specific PMCA4b expression for overall survival after pulmonary metastasectomy (*p* = 0.437).

Metastases with considerable anaplasticity, however, demonstrated a significantly lower PMCA4 expression. Nevertheless, anaplasticity and PMCA4 IHC scores showed no correlation with overall survival after metastasectomy (Figure 6).

### 2.6. PMCA4 mRNA Levels Are Prognostic for Overall Survival after PD1 Blockade Immune Checkpoint Inhibition Therapy

**Figure 7:**
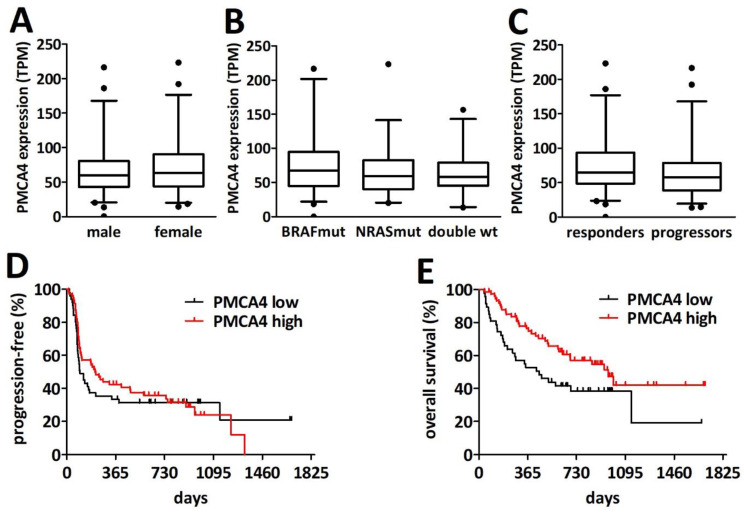
PMCA4 is a prognostic factor in PD-1 inhibitor treated melanoma patients. (**A**) There is no difference in the distribution of PMCA4 transcript levels between male (n = 71) and female (n = 50) patients (TPM—transcript per million). (**B**) There is no significant difference in PMCA4 mRNA levels between BRAF mutant (n = 51), NRAS mutant (n = 34) and double wild type (n = 36) tumors (**C**) PMCA4 levels in responders (n = 65) and in patients with progressive disease (n = 56; *p* = 0.0677). (**D**) PMCA4 levels showed no significant impact on progression-free survival after PD-1 blockade. (**E**) High PMCA4 transcript levels were associated with longer overall survival (*p* = 0.0182).

To explore the association of PMCA4 expression with the response to immune checkpoint inhibitor therapy (i.e., PD1 blockade by nivolumab or pembrolizumab) we analyzed the cutaneous melanoma RNA-seq data from a previously published cohort of 121 patients [26]. We found no difference in the abundance of PMCA4 transcripts between male (n = 71) and female (n = 50) patients (Figure 7A). Furthermore, the presence of BRAF or NRAS mutations had no impact on PMCA4 transcript levels (Figure 7B). Interestingly, there was a statistically non-significant increase in PMCA4 RNA levels in the patient who responded to the PD1 blockade (Figure 7C). Of note, PMCA1 RNA levels showed no association with response and did not correlate with PMCA4 transcript levels (Supplementary Materials Figure S4). While the difference in progression-free survival was not statistically significant when dichotomized as low (n = 51) and high (n = 70) PMCA4 expressors (98 versus 201 days, *p* = 0.592), we found a significantly longer overall survival in the high (n = 74) PMCA4 level subcohort when compared to the low (n = 47) PMCA4 level subcohort (968 versus 451 days, *p* = 0.0182).

## 3. Discussion

PMCA proteins are important regulators of the intracellular Ca2+ signal. The house-keeping isoform PMCA1 is expressed in all cell types [9]. PMCA4 is also widely expressed but it is present in a high amount in the heart, endothelial cells and different types of epitheliums [27]. In the current study, we found that PMCA4 is strongly expressed in the human skin and mucosa as indicated in the *Human Protein Atlas* [28]. Our analysis suggests that PMCA4 expression is differentiation dependent and the differences in epidermal and mucosal differentiation affect the PMCA4 expression pattern. Previous studies indicated that osteoclast differentiation and spermatogenesis is associated with changes in PMCA1 and PMCA4 expression [29,30].

To the best of our knowledge, we are the first to demonstrate that nevus cells lack PMCA4 protein expression. Previous mRNA-based studies found PMCA4 transcripts in nevus specimens; however, our data suggest that it likely originates from the unavoidable epidermal contamination and not from the nevus cells themselves [31,32]. Alternatively, nevus cells might transcribe the *ATP2B4* gene but may not translate the transcripts to the PMCA4 protein.

We demonstrated that cutaneous, choroidal and conjunctival melanoma cells express PMCA4 in the plasma membrane. We also analyzed previous gene expression microarray and RNA-seq-based mRNA studies, which indicated that there is a wide range of PMCA4 transcript levels in various cutaneous melanoma specimens. Our immunohistochemistry analysis indicated that indeed the melanoma cell specific expression also has major intensity differences among the patients. By analyzing the relative abundance of the transcripts and proteins across all the samples both in the TCGA and MM500 databases [33], ATP2B4 was close to the best-fitting curve suggesting a strong correlation between transcript and protein abundance in melanoma tissues (Supplementary Materials Figure S5). Of note, keratinocytes and endothelial cells also express PMCA4 and thus the abundance of PMCA4 transcript in the RNA pool isolated from melanoma tissue does not only depend on melanoma cell specific expression. Nevertheless, the lack of PMCA4 expression in nevi raises the possibility that PMCA4 immunohistochemistry might contribute to the sometimes diagnostically challenging differentiation of nevus and melanoma and further comparative studies are warranted.

In colon cancer, PMCA4 protein expression is decreasing during the malignant transformation and progression [12]. The prognostic relevance of PMCA4 expression has already been studied in a limited number of tumor types and it appears to be tumor type specific. In gastric cancer, a low PMCA4 immunohistochemistry score is associated with advanced TNM stage and poor prognosis [34]. In pancreatic cancer, PMCA4 is overexpressed in tumor cells and high PMCA4 expression is associated with poor survival [13]. In our study, the progression-free survival of female patients in stage I–III primary melanoma showed a significant association with PMCA4 transcript levels, suggesting that PMCA4 as well might suppress the progression of the diseases. Of note, there was a strong tendency for longer overall survival in this comparison. Surprisingly, this association was limited to female patients.

It is well established that melanoma epidemiology as well as prognosis and therapeutic response has gender specific differences [35]. Men are more prone to develop melanoma [36]. There are several potential factors that may contribute to this effect such as differences in sex hormone production, sun exposure and immunological responses [37]. Previous studies also indicated that the overall survival of male patients after pulmonary metastasectomy is shorter when compared to female patients [25]. In contrast, it seems that male patients have a greater benefit from immune checkpoint inhibitor therapies [38]. In the current analysis of the TCGA cohort there was no difference in progression-free and overall survival between female and male patients. Similarly, we found no gender related survival differences in the PD1 blockade treated cohort.

Regarding immune checkpoint inhibition, we found a strong tendency for increased PMCA4 transcript levels in patients who respond to PD1 blockade when compared to patients with progressive disease. Furthermore, patients with high PMCA4 levels had a significantly longer overall—but not progression-free—survival. These findings suggest that PMCA4 may carry predictive information for ICI response. From an immunological point of view, PMCA proteins not only influence the tumor cell Ca^2+^ homeostasis, but are also critical regulators of T-cell function. PMCA1 and PMCA4 isoforms are the major extrusion mechanisms in T cells and they play an important role in the activation and differentiation of T cells [39]. PMCAs form heteromeric complexes with either of the two Ig domain-containing proteins basigin (CD147) or neuroplastin and this interaction is required for the stability and proper localization of the PMCA proteins [40]. It was demonstrated that in murine CD4 T cells lacking neuroplastin expression of PMCAs was strongly reduced resulting in impaired calcium clearance after stimulation and increased activation of transcription factor NFAT. The sustained elevation of calcium levels in T helper cells without neuroplastin caused a bias towards Th1 polarization [41]. In human T cells, PMCA4 was shown to be an interaction partner of CD147 and PMCA4 is required for CD147-dependent inhibition of IL-2 [42].

While our study clearly demonstrates that PMCA4 expression shows a great variability in human melanoma specimens, further studies are warranted in order to explore the regulation of PMCA4 expression. Of note, there is one missense mutation of PMCA4 that is clearly associated with a case of familial spastic paraplegia [43]. Furthermore, the TCGA identified two stop-gained and 24 missense mutations from 470 melanoma cases. However, none of the mutations are recurring and have known clinical significance and they are almost all unique in the entire COSMIC database across all cancer types [44]. Thus, somatic mutations in the ATP2B4 gene are unlikely to explain the differences in PMCA4 expression.

Our previous preclinical studies indicated a metastasis suppressor function of PMCA4 in melanoma [14,15,16,17]. Our in silico analysis of a recent large cell line panel with in vivo metastatic potential screen corroborates the putative metastasis suppressor function of PMCA4 [45]. By injecting 11 different barcoded melanoma cell lines into the heart of immunocompromised mice the overall metastatic potential showed a significant inverse correlation with PMCA4 mRNA levels in the melanoma cell lines (Supplementary Materials Figure S6). Finally, this current study including several clinical cohorts further supports the notion that differences in PMCA4 expression are associated with the progression and outcome of human melanoma.

## 4. Methods

### 4.1. Patient Cohorts

In total, 26 nevus and 32 primary melanoma material was selected from tissues that were diagnosed between 2011 and 2021 at the 2nd Department of Pathology, Semmelweis University in order to represent the major types and various stages of the lesions. Primary melanoma pathological T stage was determined according to the contemporary UICC staging (7th or 8th edition).

The melanoma lung metastasectomies were performed between 2010 and 2016 at the Department of Thoracic Surgery and Thoracic Endoscopy, University Medicine Essen—Ruhrlandklinik. The case series was previously published [25], 13 cases had to be excluded due to the lack of sufficient tumor material for immunohistochemistry.

For transcript level analysis, we investigated two cohorts including 424 stage I–III patients from the TCGA cutaneous melanoma database and 121 ICI treated patients from the cohort previously published in [26]. Whole transcriptome sequencing for the ICI treated cohort was performed as described in [26]. We extracted the clinicopathological and ATP2B4 expression (TPM) data and performed the statistical comparisons.

### 4.2. Immunohistochemistry

A total of 3 µm sections from formalin fixed and paraffin embedded tissues were stained for PMCA4 (JA9 clone, AB2783, Abcam) on the Ventana BenchMark Ultra automated staining system (Roche Tissue Diagnostics, Grenzach-Wyhlen, Germany). Antibody binding was detected with the UltraVision LP Detection System (Lab Vision Corporation) which was used for antibody binding detection. Color development with the OptiView staining kit (Roche Tissue Diagnostics, Grenzach-Wyhlen, Germany) was followed by hematoxylin counterstaining. In primary melanoma, the cellular morphology was described as epitheloid or spindle cell or mixed. Anaplastic morphology was indicated in the melanoma lung metastatic specimens if regions with undifferentiated cellular morphology and pleomorphic nuclei were identified. An expert pathologist scored the staining. Endothelial PMCA4 expression served as an internal control. The melanoma cell specific scores were as follows: 0—no expression, 1, 2 and 3 for low, intermediate and high staining intensity. If considerable heterogeneity was present in the staining intensity, then 0.5, 1.5, and 2.5 scores were assigned.

### 4.3. Mutational Analysis

For the early cases prior to routine molecular analysis for BRAF and NRAS mutations, DNA was isolated from five 10-µm sections of formalin fixed and paraffin embedded melanoma lung metastasis specimens using the QIAamp DNA FFPE Tissue Kit (Qiagen, MD, USA) according to the manual’s instructions. DNA concentrations were measured by the Qubit^®^ 2.0 Fluorometer dsDNA HS assay kit (LifeTechnologies, CA, USA). Genetic analysis with targeted next-generation-sequencing was performed using a custom designed melanoma panel as described earlier [46].

### 4.4. Statistical Analysis

The Shapiro–Wilk normality test was used to analyze the distribution of continuous variables. PMCA4 expression was compared between two subgroups using the unpaired t-test or the non-parametric Mann–Whitney test depending on the distribution of variables. PMCA4 levels according to mutational status, stage or localization were compared with Kruskal–Wallis test. Categorical data were compared using the Fisher’s exact test and for mutational status the Chi-square test was used. OS was defined as the time from the diagnosis of melanoma for the TCGA cohort, from the first lung metastasectomy for the pulmonary metastasis cohort and from the first application of PD-1 blockade in the ICI treatment cohort until death or last follow-up for censored patients. Statistical differences in survival were assessed by the log-rank test. Cutoff values were determined as described previously [47] and OS and PFS survival plots were visualized by the Kaplan–Meier method. *p*-values are given as 2-sided and were considered significant below 0.05. All statistical analyses were performed using GraphPad Prism 5.0 (GraphPad Software, La Jolla, CA, USA).

## 5. Conclusions

In the current study, we demonstrated that PMCA4 protein is expressed in a differentiation specific manner in human skin and mucosa, while it is not present in nevus cells. Both primary and metastatic melanoma cells produce PMCA4 protein to a varying extent and PMCA4 transcript levels carry gender dependent prognostic information. Importantly, high primary cutaneous melanoma PMCA4 transcript levels are associated with longer overall survival after PD1 blockade at the advanced disease stage. These findings warrant further clinical studies of PMCA4 expression in melanoma and beyond.

## Figures and Tables

**Table 1:** Characteristics of the primary melanoma cases stained for PMCA4.

		Total (n = 32)
**Gender**	male female	16 (50%) 16 (50%)
**Age**	<60 years >60 years	12 (37.5%) 20 (62.5)%
**Site**	trunk extremities head and neck choroidal conjunctival	8 (25%) 7 (22%) 7 (22%) 7 (22%) 3 (9%)
**pT stage ** (NA = 5)	1 2 3 4	7 (28%) 6 (24% 8 (32%) 6 (24%)
**Morphology**	epitheloid spindle cell mixed	20 (62.5%) 5 (15.5%) 7 (22%)

NA—not available.

**Table 2:** Clinicopathological characteristics of the stage I–III cutaneous melanoma TCGA cohort.

		Total (n = 424)	Median PMCA4 mRNA Level	*p*-Value
**Gender**	male female	262 162	3975 4043	0.596
**Age** (NA = 5)	<60 ≥60	217 202	3991 4035	0.785
**Site** (NA = 41)	trunk extremities head and neck	156 182 45	3985 3962 4070	0.861
**Stage** (NA = 34)	I II III	80 140 170	3967 4068 3986	0.874
**Mutation**	BRAF mutation NRAS mutation Double wildtype	203 116 105	4102 4185 3629	0.142

NA—not available.

**Table 3:** Clinicopathological characteristics of the melanoma lung metastasectomy cohort.

		Total (n = 48)	PMCA4 Low (n = 19)	PMCA4 High (n = 29)	*p*-Value
**Gender**	male female	22 (46%) 26 (54%)	10 (53%) 9 (47%)	12 (41%) 17 (59%)	0.557
**Age**	<60 ≥60	24 (50%) 24 (50%)	12 (63%) 7 (37%)	12 (41%) 17 (59%)	0.238
**Anaplastic component**	yes no	27 (56%) 21 (44%)	16 (84%) 3 (16%)	11 (38%) 18 (62%)	**0.003**
**Maximum** **nodule size**	<20 mm ≥20 mm	29 (60%) 19 (40%)	11 (58%) 8 (42%)	18 (62%) 11 38%)	0.773
**Mutation**	BRAF NRAS double WT	21 (44%) 15 (31%) 12 (25%)	7 (37%) 7 (37%) 5 (26%)	14 (48%) 8 (28%) 7 (24%)	0.712

## Data Availability

Upon reasonable request, all data can be obtained from the corresponding author.

## Supplementary Materials

The supporting information can be downloaded at: https://www.mdpi.com/article/10.3390/ijms23063324/s1.
