# Supplementary material for: The Prognostic Relevance of PMCA4 Expression in Melanoma: Gender Specificity and Implications for Immune Checkpoint Inhibition
Source: Int J Mol Sci. 2022 Mar 19;23(6):3324. doi: 10.3390/ijms23063324 (PMC8949876; doi:10.3390/ijms23063324)
Supplement: Supplementary file 1 [file ijms-23-03324-s001.zip › ijms-1631141-supplementary.pdf]

<u>Supplementary material</u>

Supplemental Table S1. *Characteristics of the nevus specimens*

		Total (n=26)
**Gender**	male<br>female	7<br>19
**Age**	<40 years<br>40-60 years<br>>60 years	15<br>6<br>5
**Site**	trunk<br>extremities<br>head and neck	20<br>4<br>2
**Type**	junctional<br>compound<br>dermal<br>dysplastic	3<br>6<br>6<br>11

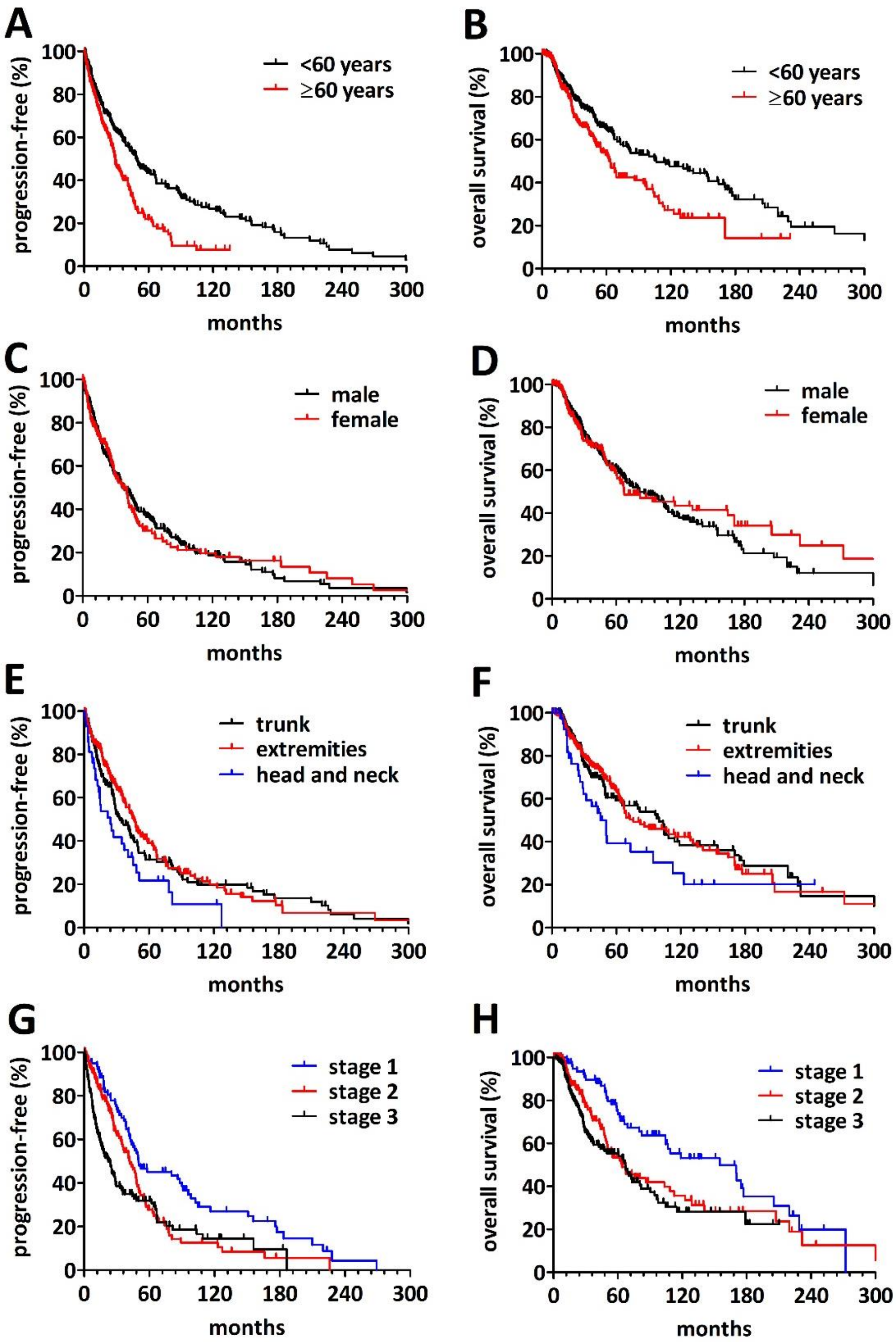

Supplemental Figure S1. *Prognostic factors in the primary cutaneous melanoma TCGA cohort. (A-B) Age is a significant prognostic factor for both progression-free and overall survival. (C-D) Gender had no impact on progression-free and overall survival. (E-F) Localization has a significant impact in progression-free survival. Furthermore, head and neck melanoma localization associates with a significantly shorter overall survival when compared to the combined trunk and extremities subcohort (p=0.028). (G-H) As expected stage I melanoma confers a better prognosis both in terms of progression-free and overall survival.*

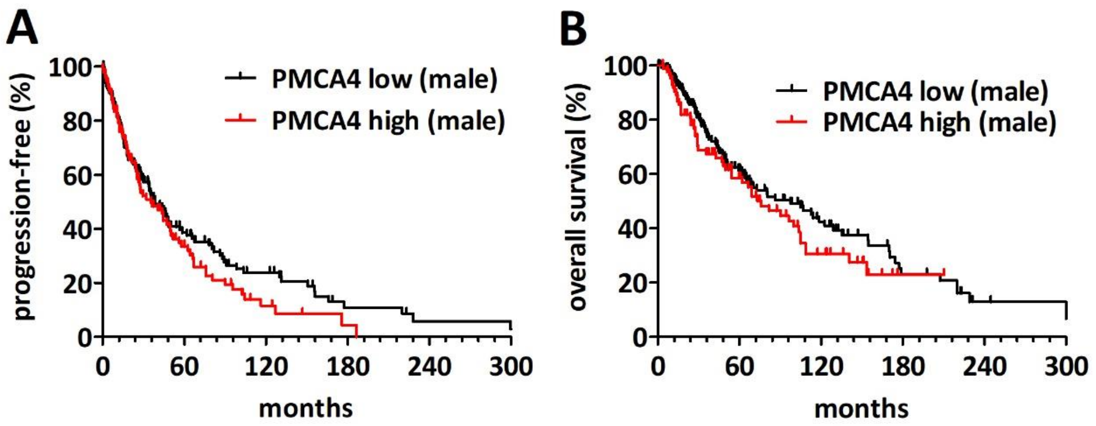

Supplemental Figure S2. *PMCA4 transcript levels in the male patients of the primary cutaneous melanoma TCGA cohort. (A) In male patients, high PMCA4 levels had no impact on progression-free survival (p=0.176). (B) There is no difference in overall survival in male patients with high or low PMCA4 mRNA levels (p=0.218).*

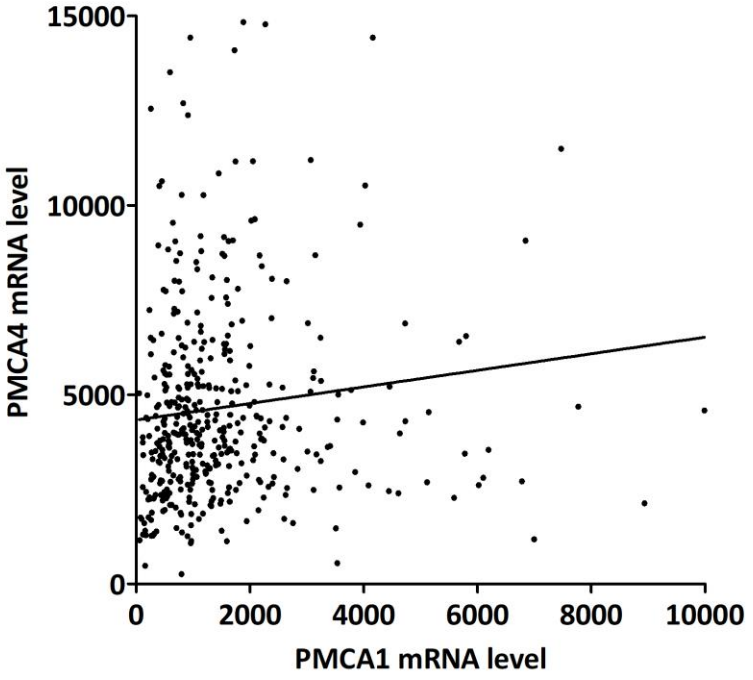

Supplemental Figure S3. *Correlation of PMCA1 and PMCA4 transcript levels in the TCGA cohort. There was a weak but significant positive correlation (Spearman r = 0.149, p=0.0021).*

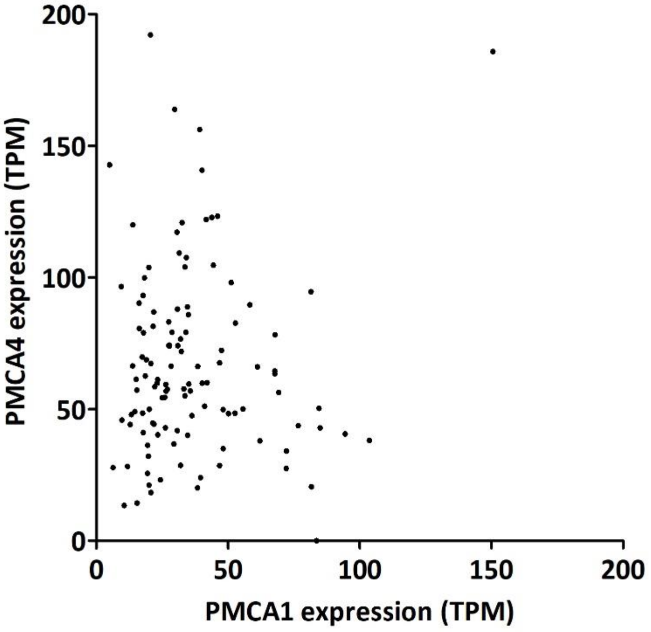

Supplemental Figure S4. *Correlation of PMCA1 and PMCA4 transcript levels in the immune checkpoint inhibitor treated cohort. There was no significant correlation (Spearman r = -0.037, p=0.686).*

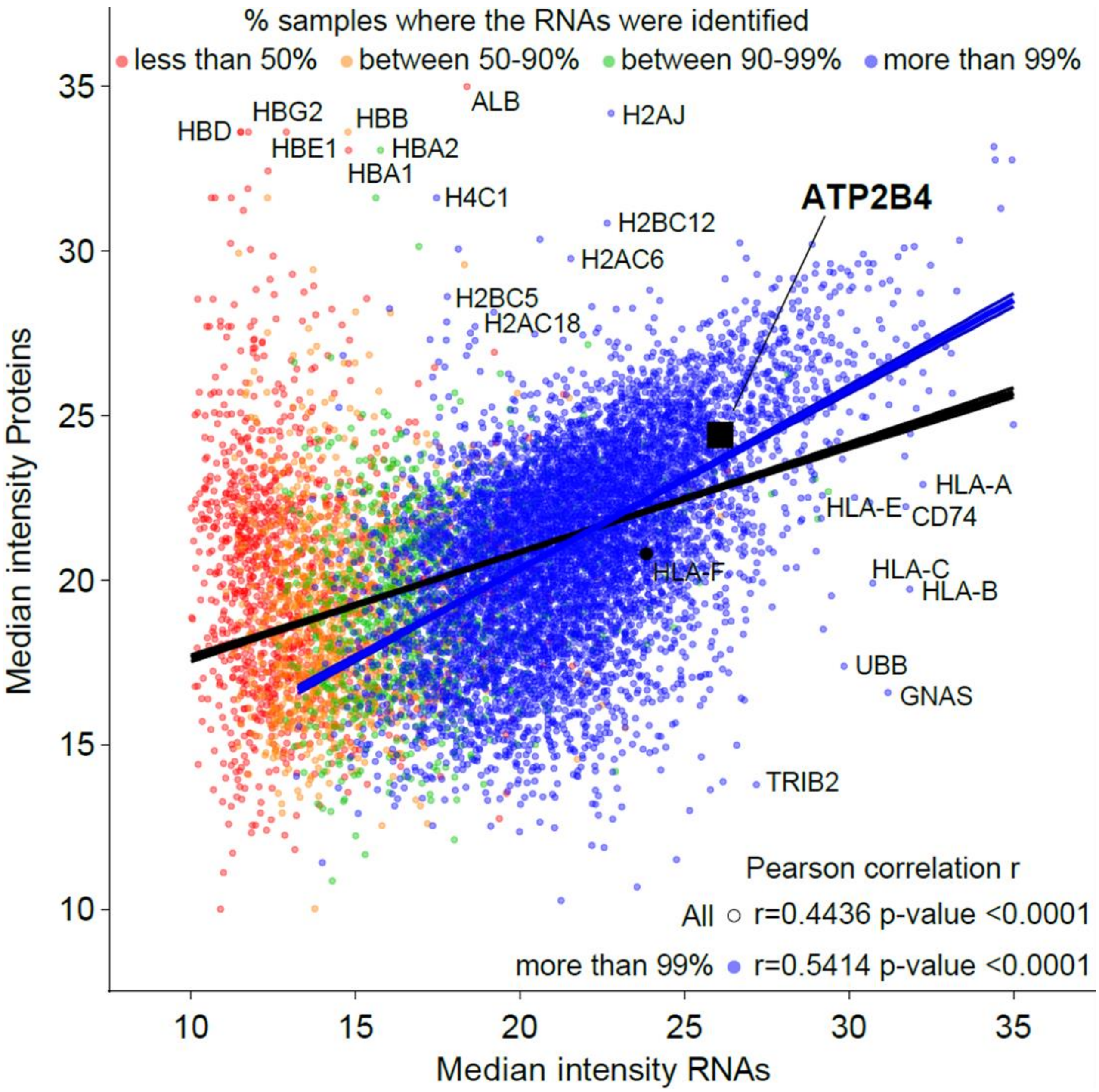

Supplemental Figure S5. *Correlation between mRNA and protein expressions in the MM500 melanoma proteome and TCGA melanoma transcriptome. The relative abundance of the transcripts and proteins was calculated based on the mean across all the samples both in the TCGA and MM500 databases, respectively. ATP2B4 was close to the best-fitting curve for the transcripts that were quantified in more than 99% of the TCGA specimens. Figure modified from Betancourt et al, 2021 [31].*

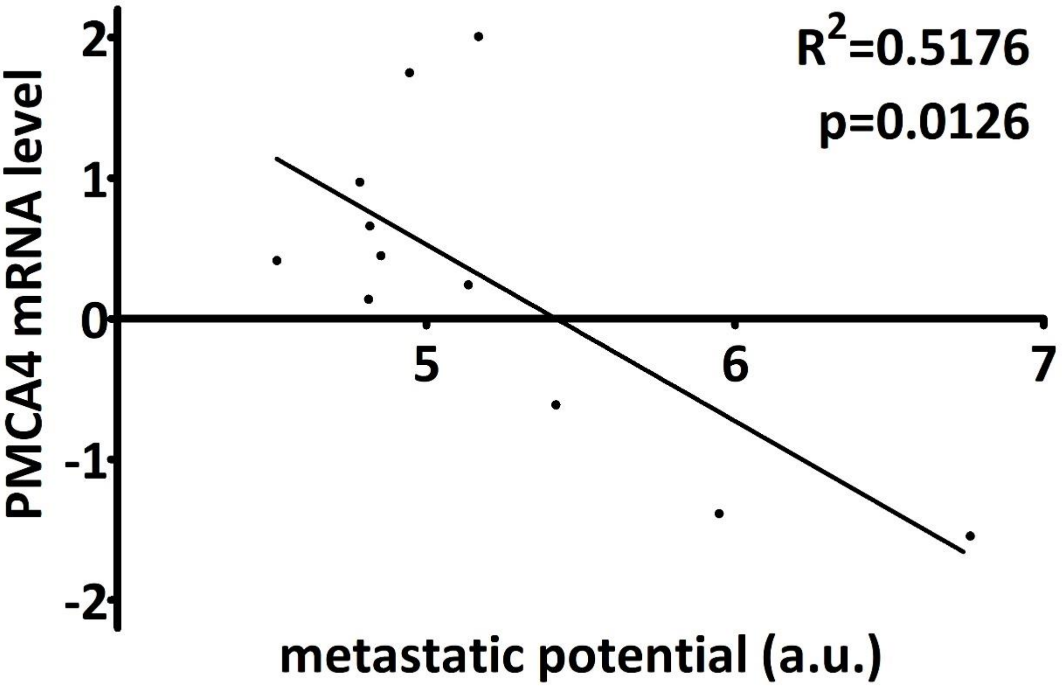

Supplemental Figure S6. *Metastatic potential and PMCA4 transcript levels for 11 melanoma cell lines from the MetDep 125 study.*
